# Identification of Linkages between EDCs in Personal Care Products and Breast Cancer through Data Integration Combined with Gene Network Analysis

**DOI:** 10.3390/ijerph14101158

**Published:** 2017-09-30

**Authors:** Hyeri Jeong, Jongwoon Kim, Youngjun Kim

**Affiliations:** 1 Environmental Safety Group, Korea Institute of Science and Technology (KIST) Europe, Campus E 7.1, Saarbruecken D-66123, Germany; h.jeong@kist-europe.de (H.J.); youngjunkim@kist-europe.de (Y.K.); 2 Division of Energy and Environment Technology, KIST School, University of Science and Technology, Hwarang-ro 14-gil 5, Seoul 02792, Korea

**Keywords:** endocrine disrupting chemicals, personal care products, breast cancer, data integration, gene network analysis

## Abstract

Approximately 1000 chemicals have been reported to possibly have endocrine disrupting effects, some of which are used in consumer products, such as personal care products (PCPs) and cosmetics. We conducted data integration combined with gene network analysis to: (i) identify causal molecular mechanisms between endocrine disrupting chemicals (EDCs) used in PCPs and breast cancer; and (ii) screen candidate EDCs associated with breast cancer. Among EDCs used in PCPs, four EDCs having correlation with breast cancer were selected, and we curated 27 common interacting genes between those EDCs and breast cancer to perform the gene network analysis. Based on the gene network analysis, ESR1, TP53, NCOA1, AKT1, and BCL6 were found to be key genes to demonstrate the molecular mechanisms of EDCs in the development of breast cancer. Using GeneMANIA, we additionally predicted 20 genes which could interact with the 27 common genes. In total, 47 genes combining the common and predicted genes were functionally grouped with the gene ontology and KEGG pathway terms. With those genes, we finally screened candidate EDCs for their potential to increase breast cancer risk. This study highlights that our approach can provide insights to understand mechanisms of breast cancer and identify potential EDCs which are in association with breast cancer.

## 1. Introduction

The rapid growth of the chemical industry and technology has led to the production of a huge amount of synthetic chemicals over the last century. It is estimated that one million metric tons of pesticides have been applied every year [1] and global plastic production was 322 million metric tons in 2015 [2]. Chemicals can be found in diverse sources such as industrial waste, soil, or ground water contaminated by pesticides, food and beverage containers, cosmetics, and personal care products (PCPs) [3]. In the past decades, there have been studies that have indicated that commercially produced synthetic chemicals can impede the actions of hormones [4] and may lead to adverse health effects, e.g., infertility, cancerous tumors, and birth defects [5]. These chemical pollutants are well known to be endocrine disrupting chemicals (EDCs) and their extensive distribution in the environment and in humans has emerged as a significant worldwide issue [6,7].

EDCs are defined as follows: “*an endocrine disruptor is an exogenous substance or a mixture that alters function(s) of the endocrine system and consequently causes adverse health effects in an intact organism, or its progeny, or (sub) populations*” by the International Program on Chemical Safety [6,8]. Approximately 1000 chemicals have been reported to have endocrine effects [9]. Human exposure monitoring data showed that EDCs can be detected in human serum, breast milk, and urine samples, as well as in placenta [10,11]. EDCs can be found in consumer products, such as PCPs [12]. PCPs frequently contain chemical additives used as preservatives, fragrances, and disinfectants to maintain and improve the function of PCPs, such as cleansing and sun protection products, or perfumes. From in vivo, in vitro, and clinical studies, some chemical ingredients such as parabens, polycyclic musks, salicylates, and phthalate esters in PCPs were revealed to induce estrogenic effects [12,13,14,15]. Such epidemiologic case studies found that exposure to estrogen-mimicking EDCs from consumer products can change serum hormone concentrations. Long-term and combined exposure to estrogen-mimicking EDCs can cause detrimental biological effects, even at low levels of effective concentrations [16,17,18].

Estrogen is considered as a main etiological factor in breast cancer [19], which can induce carcinogenic effects via three major mechanisms [20]. First, estrogen can stimulate the proliferation of breast epithelium through its binding to estrogen receptor (ER). Second, estrogen or its metabolites can exert direct genotoxic effects. Third, it can induce aneuploidy [20,21,22,23]. Breast cancer can be classified into ER-positive or ER-negative breast cancer depending on ER expression in tumor cells. Approximately 70% of breast cancers are ER-positive [24], meaning the cancer cells can respond to estrogen. Unopposed estrogen or estrogen-mimicking EDCs can bind to ER, which then alters expression of estrogen-responsive genes related to cell proliferation or apoptosis [21,25]. Several estrogen-mimicking EDCs, such as bisphenol A and phthalates, have been reported to derive epigenetic modification or genotoxic effects [26,27,28], which can modulate gene products and relevant pathways involved in certain diseases as well as breast cancer. However, adequate studies are insufficient to investigate causal mechanisms between breast cancer and estrogen-mimicking EDCs [6,8]. Due to the fact that the mechanisms remain unclear, screening for EDCs causing breast cancer is a challenging task [8]. Identifying EDC responsive genes and their relevant pathways may offer clues for finding potential EDCs and understanding how EDCs affect the development of breast cancer.

Advancements in high throughput ‘omics’ data technologies have enabled the analysis of large genomic datasets [29]. Various bioinformatics tools and public databases have been established over the last few decades, and are frequently used in the analysis of ‘omics’ data. For instance, bioinformatics enrichment tools (e.g., the database for annotation, visualization and integrated discovery (DAVID), GOrilla, EasyGO, and GO-Elite, etc.) [30,31,32,33] can be applied to analyze large numbers of gene lists and for biological interpretation. Public databases, including the Kyoto encyclopedia of genes and genomes (KEGG) and Reactome, provide information regarding genomic and metabolic networks. These databases can be integrated into analysis tools to help researchers gain a comprehensive understanding, and to aid in the identification of more efficient approaches for dealing with a large volume of datasets [29,34]. Several studies have recently applied these systemic approaches. For example, He et al. [35] examined key genes and pathways in hepatocellular carcinoma transformed from cirrhosis using bioinformatics analysis. Another study identified potential environmental chemical-interacting genes that may play important roles in the development of glioblastoma using bioinformatics tools [36]. Roy et al. [37] integrated bioinformatics data from the comparative toxicogenomics database (CTD) and KEGG and analyzed the list of genes using the gene networking tools to assess the relationship between EDCs exposure and disease development. Further, Vlasblom et al. [38] revealed novel functions for uncharacterized genes in *E. coli* using the gene network analysis tool, GeneMANIA. However, only a few studies considered identifying the effects of exposure to EDCs by combining bioinformatics data and gene network analysis.

We thus hypothesized that a systemic bioinformatics approach can integrate dispersed data to derive linkages between EDCs and breast cancer. In the present study, we aimed to (i) understand causal molecular mechanisms between EDCs used in PCPs and ER-positive breast cancer; and (ii) identify candidate EDCs associated with breast cancer by conducting data integration combined with gene network analysis using bioinformatic tools and public biological databases.

## 2. Methods

**Figure 1:**
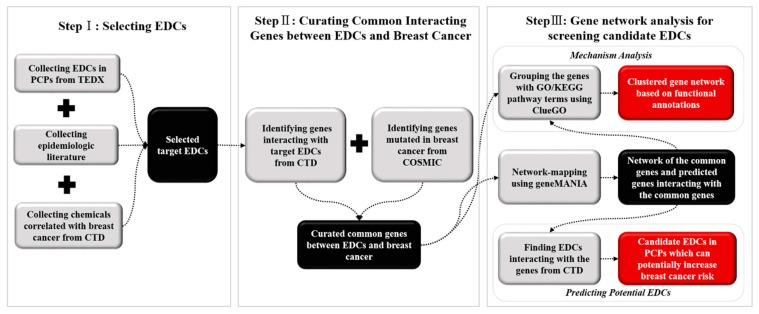
Scheme of this study including the data integration combined with gene network analysis to identify associations between endocrine disrupting chemicals (EDCs) and breast cancer. Notes: TEDX—The endocrine disrupting exchange database; CTD—Comparative toxicogenomics database; COSMIC—Catalogue of somatic mutations in cancer database; GO—Gene Ontology; KEGG—Kyoto encyclopedia of genes and genomes.

The study was basically conducted in three steps: (i) selecting EDCs for which evidences exists that exposure to them may result in breast cancer and collecting epidemiologic data from previously published literature; (ii) identifying the common genes associated with both EDCs and ER-positive breast cancer; and (iii) visualizing gene networks and identifying their related biological pathways as well as predicting candidate EDCs which can potentially increase breast cancer risk. Figure 1 illustrates the workflow, resources, and bioinformatic tools used in this study.

### 2.1. Data Collection and EDC Selection

The endocrine disruption exchange (TEDX) database was used to investigate the EDCs found in PCPs and cosmetics [39]. TEDX classified a chemical as an EDC if it has at least one study demonstrating endocrine disrupting properties in the published data. The EDCs listed in the ‘PCP/Cosmetic ingredient’ category in TEDX were searched via CTD [40] to find their correlations with breast cancer. CTD was developed by the Mount Desert Island Biological Laboratory (MDIBL), supported by the National Institute of Health (NIH), and integrates information about chemicals, diseases, and genes and curates their associations [41]. The EDCs in this study were finally selected by reviewing published epidemiologic studies. Figure 1 briefly shows the study steps.

### 2.2. Curating Common Interacting Genes between EDCs and Breast Cancer

EDC-interacting genes in *Homo sapiens* were extracted from CTD. In CTD, chemical–gene interactions in vertebrates and invertebrates are manually curated from the published literature acquired from PubMed. Interaction types are classified using a hierarchical interaction-type vocabulary that characterizes physical, regulatory, and biochemical interactions between chemicals and genes [42]. The genes that are mutated in ER-positive breast cancer were downloaded from the cancer browser available in the catalogue of somatic mutations in cancer (COSMIC) [43], which is a database of somatic mutations and relevant information for human cancer [44]. ‘Breast’ and ‘Include all tissues’ were selected for tissue selection, and ‘Carcinoma’ and ‘ER-positive carcinoma’ were chosen for histology selection. Next, specific genes that overlapped between mutated genes in ER-positive breast cancer and EDC-interacting genes were curated using the MyVenn tool in CTD.

### 2.3. Gene Nework Analysis and Screening Potential EDCs

GeneMANIA (University of Toronto, Toronto, ON, Canada), a plugin which can be installed from ‘App Manager’ in cytoscape, was applied to conduct a network analysis of the common genes and to predict related genes. Cytoscape (Institute of Systems Biology, Seattle, WA, USA) is an open source software for visualizing and analyzing biological networks [45,46] that can be downloaded from the cytoscape website [47]. Plugins are available from cytoscape, which provide additional network analyses to extend its functionality [45]. GeneMANIA analyzes the networks of a set of input genes and predicts genes with their categorized functional association implied by genomic and proteomic data sources such as the biological general repository for interaction datasets (BioGRID), gene expression omnibus (GEO), interologous interaction database (I2D), and Pathway Commons. The networks are grouped into six categories: co-expression, co-localization, genetic interaction, physical interaction, and predicted and shared protein domains. We predicted the associated genes and their interaction networks using the GeneMANIA algorithms. The algorithms consist of two parts: a linear regression-based algorithm that calculates a single composite functional association network based on multiple data sources; and a label propagation algorithm that was used to predict gene function given the composite functional association network. Predicted genes were scored based on their relevance to the original query genes. For details, see references [48,49]. The *Homo sapiens* database of GeneMANIA was first downloaded (updated on 17 March 2017), and then the list of 27 common genes curated in Section 2.2 was imported. The degree of centrality, which is the number of links that a node has in the network [50], was identified for each of the 27 input genes based on the results of network mapping by GeneMANIA. ClueGO (INSERM, Paris, France) is another cytoscape plugin developed to facilitate the biological interpretation and visualization of functional annotations with a hypergeometric test corresponding to a list of genes [51]. In the present study, ClueGO v2.3.3 was used to functionally group input genes and newly predicted genes by their associated KEGG pathway and gene ontology (GO). *p* value of less than 0.05 and kappa coefficient of 0.4 were set as threshold values. The common genes were searched via CTD to find potential candidate EDCs, excluding the selected EDCs from the first step. A search with the genes in CTD found interacting chemicals having in vitro chemical-gene interacting data in *Homo sapiens*, and among these chemicals, chemicals which are included in the ‘PCP/Cosmetic ingredient’ category in TEDX were extracted as candidate EDCs. We calculated an individual score of each candidate EDC for breast cancer to derive priority candidates with considering the degree centrality of the genes interacting with the EDCs. The score was calculated in the sum of the degree centrality of related nodes representing genes which could interact with the EDCs. This is due to the fact that nodes with higher degree are more central in the network.

## 3. Results

### 3.1. Data Integration

Of the 117 EDCs in TEDX, 88 EDCs had chemical information included in CTD. Six EDCs: caffeine, apigenin, captan, diethylhexyl phthalate (DEHP), diethyl phthalate (DEP), and lindane, were selected. These were curated by CTD as either correlates with breast cancer or because they may play a role in the etiology of breast cancer. Caffeine and apigenin were excluded because the relationship between these two substances and breast cancer in epidemiologic studies was inconsistent [52,53,54,55,56]. There is little evidence that DEHP increases risk of breast cancer so far. However, phthalate congeners have been reported to be xenoestrogens, and may contribute to the development of breast cancer [37,57,58]. Therefore, ultimately four EDCs: captan, DEHP, DEP, and lindane, were selected for the study. The selected EDCs and their interacting genes are listed in Table 1.

GSTP1, CYP3A4, ESR1, NR1T2, and TSC22D1 are considered to interact with captan. These genes are associated with the following pathways based on annotation in KEGG: the metabolism of xenobiotics by cytochrome P450 (KEGG: 00980), and drug metabolism (KEGG: 00932). Captan is used as a pesticide, fungicide, and preservative in PCPs and cosmetics. Engel L.S et al. [59] conducted a large cohort study to examine the association between pesticide use, including captan, and breast cancer incidence among farmers’ wives. They found that the husbands’ use was associated with an increased breast cancer risk in their wives (rate ratio = 2.7, 95% confidence interval (CI) 1.7, 4.3).

DEHP was contained in cosmetics, liquid soap, detergent, and decorative inks [60]. Although the U.S. Consumer Product Safety Improvement Act (CPSIA) has banned DEHP in concentrations of more than 0.1% in products for children [61], DEHP is still widely used as plasticizer and may be found in cosmetics as leachates from packaging [62]. 115 genes are considered to relate to DEHP in *Homo sapiens* via CTD search, which are mainly involved in pathways in cancers (KEGG:05200), prostate cancer (KEGG:05215), bladder cancer (KEGG:05219), colorectal cancer (KEGG:05210), and thyroid cancer (KEGG:05216). However, there exist only few epidemiologic studies providing evidence that DEHP increases the risk of breast cancer. Lopez-Carrillo et al. [63] found that DEHP metabolites had no association with breast cancer in their study.

DEP is used in 28–71% of the PCPs, 57–72% of perfumes, and 25% of deodorants [63]. Twenty genes interact with DEP, and are mainly involved in the PPAR signaling pathway (KEGG:03320), pathways in cancer (KEGG:05200), thyroid cancer (KEGG:05216), the adipocytokine signaling pathway (KEGG:04920), and non-small cell lung cancer (KEGG:05223). Lopez-Carrillo et al. [63] examined the association between exposure to phthalates and breast cancer incidence in Mexican women. They reported that exposure to DEP is significantly associated with an increased risk of breast cancer (odds ratio (OR) = 2.20, 95% CI 1.33, 3.63).

Lindane, also referred to as gamma-hexachlorocyclohexane, has been used as an insecticide and as a medication for the treatment of lice and scabies when formulated into shampoos, lotions, and creams. 134 genes were found to interact with lindane, and its major enriched pathways are bladder cancer (KEGG:05219), pathways in cancer (KEGG:05200), steroid hormone biosynthesis (KEGG:00140), the ErbB signaling pathway (KEGG:04012), and prostate cancer (KEGG:05215). Ibarluzea et al. [64] conducted a case-control study of females in southern Spain to determine the effects of environmental estrogen on breast cancer. They found that lindane may increase the risk of breast cancer among postmenopausal women (OR = 1.76, 95% CI 1.04, 2.98).

### 3.2. Finding EDC-Interacting Genes and Mutated Genes in ER Positive Breast Cancer

**Figure 2:**
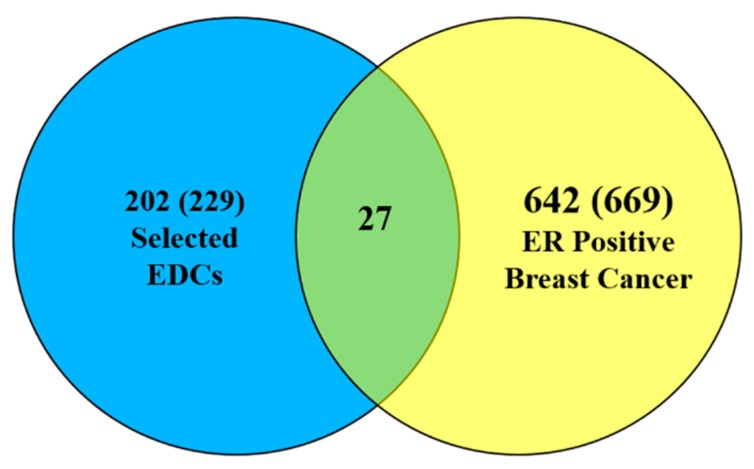
A Venn diagram of the list of genes common between ER-positive breast cancer and the four EDCs.

We searched for mutated genes in ER positive breast cancer and EDC-related genes in the COSMIC database and the CTD. 669 genes are mutated in ER positive breast cancer and 229 genes are related to the four EDCs in *Homo sapiens* based on searches of CTD (searched on December, 2016). Figure 2 is a Venn diagram of EDC interacting genes and mutated genes in ER-positive breast cancer; there are 27 overlapped genes.

### 3.3. Network Analysis of Curated Genes and Potential EDCs

**Figure 3:**
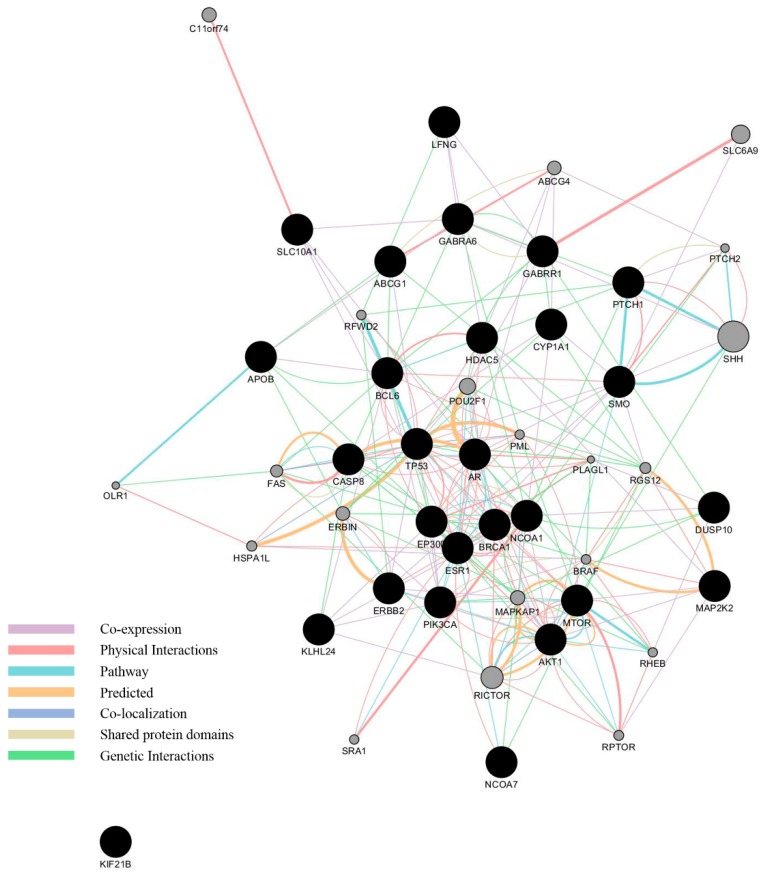
Network of the 27 common genes (black nodes) and the additional 20 predicted associated genes (gray nodes) by GeneMANIA. The node size of predicted genes represents the relevance of each gene to the 27 genes.

Using GeneMANIA, we performed a network analysis of the common genes. In addition, further 20 genes—SHH, RICTOR, SLC6A9, POU2F1, MAPKAP1, C11orf74, ERBIN, ABCG4, FAS, RGS12, HSPA1L, RPTOR, BRAF, RFWD2, SRA1, PML, RHEB, PTCH2, OLR1, and PLAGL1—were predicted to be related to the list of 27 genes. A network map of these genes is displayed in Figure 3.

ESR1 has the highest centrality, the number of links connected with other genes, followed by TP53, NCOA1, AKT1, and BCL6. Of the six categorized networks, physical interactions between the gene products were the most frequent links. Table 2 shows the top five genes with the highest centrality and interacting genes with corresponding networks. For all 27 genes, see Supplementary Materials Table S1.

**Figure 4:**
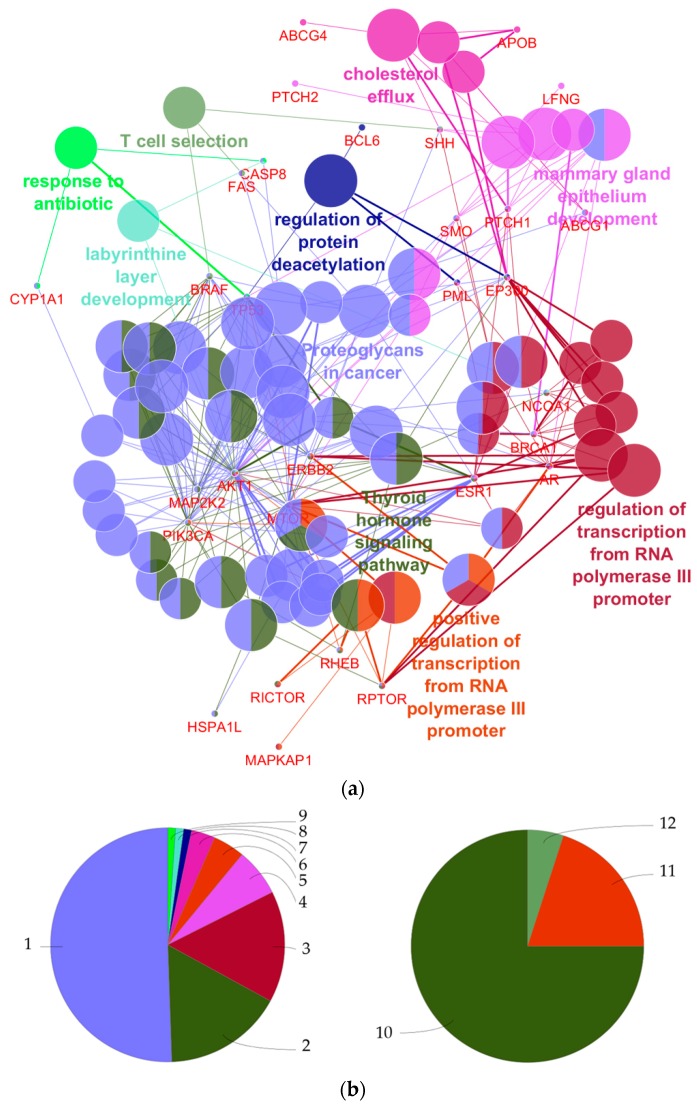
Results of visualizing functional annotations corresponding to the 47 genes using ClueGO: (**a**) Functionally grouped network with GO/KEGG terms as nodes linked based on kappa score level ≥0.4, where the label of the most significant term per group is shown only. The node size indicates the term enrichment significance; (**b**) Overview charts showing the proportion of each group associated with the 27 common genes (left side) and the 20 predicted genes (right side). 1—Proteoglycans in cancer; 2—Thyroid hormone signaling pathway; 3—Regulation of transcription from RNA polymerase III promoter; 4—Mammary gland epithelium development; 5—Positive regulation of transcription from RNA polymerase III promoter; 6—Cholesterol efflux; 7—Regulation of protein deacetylation; 8—Labyrinthine layer development; 9—Response to antibiotics; 10—Positive regulation of TOR signaling; 11—TOR complex; 12—T cell selection.

Among EDCs listed in ‘PCP/Cosmetic Category’ in TEDX, perfluorooctanoic acid, stearic acid, triphenyl phosphate, dibutyl phthalate, and sodium fluoride were predicted as the top five candidate EDCs associated with breast cancer (Table 3). The rest of the candidate EDCs are presented in Supplementary Materials Table S2. The most significant GO/KEGG pathway terms of the 27 genes which predicted the 20 genes were ‘Proteoglycans in cancer’, ‘Thyroid hormone signaling pathway’, ‘Regulation of transcription from RNA polymerase III promoter’, ‘Mammary gland epithelium development’, ‘Positive regulation of transcription from RNA polymerase III promoter’, ‘Cholesterol efflux’, ‘Regulation of protein deacetylation’, ‘T cell selection’, ‘Labyrinthine layer development’, and ‘Response to antibiotics’. (Figure 4 and Table 4).

## 4. Discussion

### 4.1. What Can Be Derived from the Functional Annotations of the 27 Genes with Regard to Breast Cancer Development?

In our study, we aimed to identify the molecular linkages between the EDCs used in PCPs and cosmetics and the development of ER-positive breast cancer. Using data integration, 27 genes that were found to be common between EDC-interacting genes and mutated genes in ER-positive breast cancer, and these genes were used as input data to perform a network analysis. Twenty further genes were predicted to be related to the 27 overlapping genes. ESR1 had the highest degree centrality among the 27 common genes. It means that ESR1 has the highest connections with other genes. This gene encodes an estrogen receptor alpha which is a nuclear transcription factor that induce cellular proliferation and differentiation. Jeselsohn et al. [65] found that the coding sequence of ESR1 gene was mutated with 12% frequency in ER-positive breast cancer. Exposure to DEHP induced to decrease ESR1 gene expression [66], and butylbenzyl phthalate altered ESR1 mRNA expression by the demethylation in the promoter region of the gene [67]. ESR1 can be suggested as a key gene to investigate the mechanisms of breast cancer cause by EDCs.

‘Proteoglycans in cancer’ was regarded as the most significant pathway/GO term associated with the common genes from the analysis conducted with ClueGO. Proteoglycans, such as hyaluronan (HA) which is known to be a major component of the extracellular matrix, are considered to be key macromolecules in the tumor microenvironment that have multiple roles in cancer progress [68]. For example, HA is synthesized and accumulated to a greater degree by invasive breast cancer cells than normal tissue [69]. HA can activate CD44, which may indirectly affect the activation of PIK3CA and phosphorylation of AKT1. These genes are reported to be related to the Phosphatidylinositol 3-kinase (PI3K)-AKT pathway, and aberrations in this pathway are common in human cancer [70,71].

From our results, AKT1, EP300, ESR1, MAP2K2, MTOR, NCOA1, PIK3CA, RHEB, and TP53 are considered to be involved in the ‘thyroid hormone signaling pathway’. Hall et al. [72] investigated the response of human breast cancer cells to thyroid hormones. They found that thyroid hormones promote proliferation of breast cancer cells and that there is an interaction between 17β-estradiol, hormonal risk factor in breast cancer, and thyroid hormone signaling mechanisms [73]. Therefore, our results seem to be in line with the findings of these previous studies and suggest that there is a molecular association between the development of breast cancer and EDCs.

### 4.2. How Can Other Potential Diseases be Predicted Based on the 20 Predicted Genes?

The 20 genes predicted using GeneMANIA are expected to be functionally associated with positive regulation of the target of rapamycin (TOR) signaling, the TOR complex, and T cell selection (Figure 4). TOR is a conserved serine/threonine protein kinase, which is called mTOR (mammalian TOR or mechanistic TOR) in mammals. mTOR regulates cellular response and metabolism in response to environmental stress [74]. For example, Wang et al. [75] found that dibutyl phthalate (DBP), a recognized EDC, may affect the mTOR signaling pathway in the cell. mTOR deregulation occurs in various human diseases, including cancer, metabolic disorders, and neurodegeneration [76,77]. Different signaling pathways can often have an influence on other pathways [78]. This phenomenon, called ‘Crosstalk’, can be seen between Ras-extracellular signal-regulated kinase (Ras-ERK) and PI3K-mTOR signaling pathways [79]. Ras-ERK signaling pathway is the major mechanism for cell survival, proliferation, and metabolism, which is frequently over-activated in tumors [80]. Regarding previous studies, we suggest that the predicted genes may be associated with not only breast cancer, but also different types of cancers, and other diseases (e.g., metabolic disorders), but further studies are needed.

The Sonic hedgehog (SHH) gene was predicted to be the gene most relevant to the 27 input genes from the GeneMANIA prediction algorithm. The hedgehog gene encodes a protein that is important in patterning the early embryo. There are three mammalian homologues of hedgehog and SHH is one of them. SHH has been reported to play critical roles in cell differentiation, proliferation at specific embryonic developmental stages, and in maintaining homeostasis in adults [81]. Decreased mRNA levels of SHH were observed after prenatal exposure to DBP in male rats [82,83]. Inactivation of the SHH gene during sexual differentiation can lead to feminization of the genital urinary tracts, such as reduced anogenital distance and hypospadias in males [84,85]. It was shown that some EDCs increased the risk of reproductive abnormalities [86,87,88]. The results of our study may suggest that EDCs causing breast cancer may also affect the reproductive system.

### 4.3. Contributions and Limitations of the Study

The top five genes with the highest degree of centrality were estimated by applying the gene network analysis based on the 27 genes in common between the selected four EDCs and ER-positive breast cancer. This finding suggests potential priority genes for designing a strategic experimental study to further investigate the genomic effect of EDCs in the development of breast cancer. In addition, the 20 genes which can directly interact with the 27 common genes were predicted in this study so that those predicted genes could be suggested to be associated with an increased risk of breast cancer as well as other diseases for further studies. We also predicted candidate EDCs based on the 47 genes (27 common genes and 20 predicted genes) and prioritized them according to their score. The higher score reflects that a candidate EDC interacts with more genes and those genes have more connection with other genes. A candidate EDC with a high score may be predicted to involve in similar biological pathways with the 47 genes, but further research is necessary. Screening EDCs from among the chemicals in consumer products is one of the main challenges [6], especially when only a limited amount of toxicological data is available. A few EDCs have been studied to assess their influence on breast cancer, but most potential EDCs have been substantially untested. This study can help select candidate EDCs which have the potential to contribute to breast cancer or other diseases. The data integration in combination with gene network analysis approach proposed in this study could show a good possibility to provide research insights as a requisite step to identify potential toxicants which are in association with their specific adverse effects at the molecular level.

There are three limitations in the present study. First, many mutated genes discovered in cancer cells are passengers that do not contribute to oncogenesis [89]. Twenty of the 27 common genes have published data presenting that these genes were associated with breast cancer (Supplementary Materials Table S3), but seven genes (DUSP10, GABRA6, GABRR1, KIF21B, KLHL24, MAP2K2, and SLC10A1) might be passenger genes or have not yet been studied regarding their pathological roles in breast cancer. However, further studies are needed to exactly distinguish genuine driver genes from passenger mutations. Second, while this study generates a hypothesis for potential involvement of EDC-interacting genes in breast cancer, further research in a laboratory setting is necessary to validate candidate EDCs and the 20 predicted genes’ role in ER-positive breast cancer. Finally, although a comprehensive set of databases on EDCs and genomic data (e.g., TEDX, CTD, and COSMIC) was used in this study, we might have missed genes or additional other potential EDCs since the entire set of literature was not considered.

## 5. Conclusions

In this study, among EDCs used in PCP, four EDCs, captan, DEHP, DEP, and lindane, having correlation with breast cancer were selected, and we curated 27 common interacting genes between those EDCs and breast cancer to perform gene network analysis. Based on the gene network analysis, ESR1, TP53, NCOA1, AKT1, and BCL6 were found to be key genes to demonstrate the molecular mechanisms of EDCs in the development of breast cancer. Using GeneMANIA, we additionally predicted 20 genes which could interact with the 27 common genes. In total, 47 genes combining the common and predicted genes were functionally grouped with the gene ontology and KEGG pathway terms. With those genes, we finally screened 52 candidate EDCs to potentially increase the breast cancer risk. Further studies are needed to validate the involvement of these EDC-interacting genes and candidate EDCs in ER-positive breast cancer. This study highlights that our approach can provide insights to uncover mechanisms of breast cancer and identify potential EDCs which are in association with breast cancer.

## Figures and Tables

**Table 1:** Four EDCs and their interacting genes searched in CTD.

Name (Cas No.)	Interacting Genes
**Captan (133-06-2)**	5 genes: GSTP1 | CYP3A4 | ESR1 | NR1I2 | TSC22D1
**Diethylhexyl Phthalate (117-81-7)**	115 genes: ABCB1 | ACADM | ACADVL | AHR | AKT1 | AMH | AOX1 | AR | ARRDC3 | BAX | BBC3 | BCL2 | CASP3 | CASP7 | CASP8 | CASP9 | CDKN1A | CDO1 | CELSR2 | CGA | CGB3 | CLDN6 | CSNK1A1 | CTNNB1 | CXCL8 | CYP19A1 | CYP1A1 | CYP1B1 | CYP2C19 | CYP2C9 | CYP3A4 | CYP4A10 | DDIT3 | DHCR24 | DIABLO | DNAJB1 | EP300 | ESR1 | ESR2 | FASN |FLG | FSHB | FSHR | GJA1 | GLI3 | GLRX2 | HDAC4 | HDAC5 | HEXA | HEXB | HMGCR | HSD11B2 | HSPA1B | ID1 | IL17RD | IL4 | KLK3 | LAMP3 | LFNG | LHCGR | LIF | MAPK1 | MAPK3 | MARS | MDM2 | MED1 | MMP2 | MMP9 | MTOR | MYC | NCOA1 | NCOR1 | NGB | NR1H3 | NR1I2 | NR1I3 | NR3C1 | NR4A1 | NR4A2 | NR4A3 | PAPSS1 | PAPSS2 |PIK3CA | PMAIP1 | PPARA | PPARB | PPARD | PPARG | PPARGC1A | PRNP | PTCH1 | PTGS2 | RPS6KB1 | RXRA | RXRB | RXRG | SCARA3 | SCD | SLC7A11 | SMO | SP3 | SQLE | SREBF1 | SREBF2 | STAR | SUOX | TIMP2 | TNF | TP53 | TSPAN6 | TXNRD1 | VCL | VEGFA | VLDLR | ZNF461
**Diethyl Phthalate (84-66-2)**	20 genes: AHR | APOA1 | APOB | AR | CASP3 | CXCL8 | CYP19A1 | CYP1B1 | ESR1 | ESR2 | FLG | NR1I2 | NR1I3 | PPARA | PPARB | PPARG | RXRA | RXRB | RXRG | SHBG
**Lindane (58-89-9)**	134 genes: ABCA1 |ABCB1 | ABCG1 | ABCG2 | ACRC | AGPAT9 | AK4 | ALDH8A1 | AR | ASNS | BAX | BCL2 | BCL6 | BIRC3 | BRCA1 | C15ORF39 | CARS | CAT | CCND1 | CCNG2 | CD69 | CD84 | CDKN1A | CEBPB | CHAC1 | CRIM1 | CX3CR1 | CYP11A1 | CYP11B1 | CYP11B2 | CYP17A1 | CYP19A1 | CYP1A1 | CYP2B6 | CYP2D6 | CYP2E1 | CYP3A4 | CYP3A7 | DAP3 |DNAJB4 | DUSP10 | ERBB2 | ERBB3 | ESR1 | ESR2 | ESRRA | EVI2A | FAM107B | FAM213B | FBXO32 | FRAT1 | GABRA1 | GABRA2 | GABRA4 | GABRA6 | GABRB1 | GABRB2 | GABRB3 | GABRG2 | GABRR1 | GCLC | GCLM | GLRA1 | GLRA2 | GLRA3 | GNRH1 | GPR18 | GPT2 | GSR | GSTM1 | HMGCS1 | HSD3B2 | HSPA1A | HYLS1 | ID1 | IER3 | IFNG | IL5 |ISL2 | JUN | KIF21B | KLHL24 | LIF | MAP2K1 | MAP2K2 | MAPK1 | MAPK3 | MMP9 | MTHFD2 | NANOS1 | NCOA7 | NFE2L3 | NOS2 | NR1I2 | NRF1 | PDCD4 | PELI1 | PGR | PLCL1 | PNRC1 | POMC | PPARGC1A | PPRC1 | RAF1 | RARA | RGS2 | RXRB | SEMA3G | SESN2 | SGK1 | SHBG | SLC10A1 | SLC22A1 | SLC3A2 | SLC7A11 | SQSTM1 | SRC | SRXN1 |STAM2 | STAR | SULT2A1 | TFAM | TFB2M | TFF1 | TM6SF1 | TMCO6 | TMEM177 | TMEM267 | TNF | TP53 | TRIB3 | VEGFA | VLDLR | ZNF628

**Table 2:** Top five genes with the highest centrality and their interacting genes and networks based on the GeneMANIA network map.

Degree Centrality	Gene	Official Full Name	Interacting Gene	Networks *
13	ESR1	Estrogen receptor 1	AKT1	2
			AR	2, 3, 5, 6
			BRCA1	2, 3
			CASP8	7
			EP300	2, 3
			ERBB2	2
			HDAC5	2
			NCOA1	2, 3
			NCOA7	2, 3
			PIK3CA	2, 3
			SLC10A1	1
			SMO	1
			TP53	2
12	TP53	Tumor protein p53	AKT1	1
			AR	2, 7
			BCL6	2, 3
			BRCA1	2, 3
			CASP8	2
			EP300	2, 7
			ERBB2	1
			ESR1	2
			HDAC5	2
			MTOR	2
			NCOA1	2
			SMO	1
12	NCOA1	Nuclear receptor coactivator 1	AKT1	1, 3
			AR	2, 3
			BRCA1	2
			CYP1A1	1
			DUSP10	1, 7
			EP300	1, 2, 3, 6
			ESR1	2, 3
			HDAC5	7
			KLHL24	1
			NCOA7	7
			PTCH1	7
			TP53	2
11	AKT1	AKT serine/threonine kinase 1	AR	2
			BRCA1	2
			EP300	2
			ERBB2	1
			ESR1	2
			MAP2K2	1
			MTOR	2, 3, 5, 4
			NCOA1	1, 3
			PIK3CA	2, 3
			SMO	3
			TP53	1
11	BCL6	B-cell CLL/lymphoma 6	ABCG1	7
			APOB	1, 7
			EP300	2, 7
			GABRR1	7
			HDAC5	2, 3
			KLHL24	1
			PIK3CA	1
			PTCH1	7
			SLC10A1	1
			SMO	2
			TP53	2, 3

* 1—Co-expression; 2—Physical interactions; 3—Pathway; 4—Predicted; 5—Co-localization; 6—Shared protein domains; 7—Genetic interaction.

**Table 3:** The list of top five candidate EDCs with the score and their interacting genes curated from CTD.

Score	Chemical Name (Cas No.)	Interacting Genes
40	Perfluorooctanoic acid (335-67-1)	ABCG1, APOB, CYP1A1, ERBB2, ESR1, TP53, SHH
37	Stearic acid (57-11-4)	ABCG1, AKT1, AR, ESR1
35	Triphenyl phosphate (115-86-6)	AR, ESR1, TP53
34	Dibutyl Phthalate (84-74-2)	AKT1, AR, ESR1
30	Sodium Fluoride (7681-49-4)	AKT1, CASP8, TP53, FAS

Note: Score = ∑Degree centrality of interacting genes.

**Table 4:** Significantly enriched KEGG pathways and biological process GO terms associated with the 27 genes common between EDCs and breast cancer and the 20 predicted genes produced by ClueGO.

GO Term	Ontology Source	*p*-Value	Annotated Genes
Proteoglycans in cancer	KEGG	4.0 × 10^−14^	AKT1|BRAF|ERBB2|ESR1|FAS|MAP2K2| MTOR|PIK3CA|PTCH1|SHH|SMO|TP53
Thyroid hormone signaling pathway	KEGG	7.2 × 10^−12^	AKT1|EP300|ESR1|MAP2K2|MTOR|NCOA1| PIK3CA|RHEB|TP53
Regulation of transcription from RNA polymerase III promoter	GO-Biological process	1.6 × 10^−8^	AR|BRCA1|ERBB2|MTOR|RPTOR
Mammary gland epithelium development	GO-Biological process	2.5 × 10^−8^	AKT1|AR|ESR1|PML|PTCH1|SMO
Positive regulation of transcription from RNA polymerase III promoter	GO-Biological process	2.8 × 10^−8^	AR|ERBB2|MTOR|RPTOR
Cholesterol efflux	GO-Biological process	5.0 × 10^−8^	ABCG1|ABCG4|APOB|PTCH1|SHH
Regulation of protein deacetylation	GO-Biological process	3.0 × 10^−6^	BCL6|EP300|PML|TP53
T cell selection	GO-Biological process	2.0 × 10^−4^	BRAF|FAS|SHH
Labyrinthine layer development	GO-Biological process	2.6 × 10^−4^	AKT1|CASP8|NCOA1
Response to antibiotic	GO-Biological process	3.4 × 10^−4^	CASP8|CYP1A1|TP53

## Supplementary Materials

The following are available online at www.mdpi.com/1660-4601/14/10/1158/s1, Table S1: Interacting genes and their network types of the 27 related genes between four selected EDCs and ER positive breast cancer based on GeneMANIA network analysis, Table S2: The list of candidate EDCs with the score and their interacting genes curated from CTD, Table S3: The common 27 genes between EDCs and breast cancer, and evidence showing their relation with breast cancer.
