# Supplementary material for: Identification of Linkages between EDCs in Personal Care Products and Breast Cancer through Data Integration Combined with Gene Network Analysis
Source: Int J Environ Res Public Health. 2017 Sep 30;14(10):1158. doi: 10.3390/ijerph14101158 (PMC5664659; doi:10.3390/ijerph14101158)
Supplement: Supplementary file 1 [file ijerph-14-01158-s001.pdf]

International Journal of
Environmental Research and Public Health

MDPI

Supplementary Materials: Identification of Linkages between EDCs in Personal Care Products and Breast Cancer through Data Integration Combined with Gene Network Analysis

Hyeri Jeong [1,2], Jongwoon Kim [1,2,*] and Youngjun Kim [1,2]

Table S1. Interacting genes and their network types of the 27 common related genes between four selected EDCs and ER positive breast cancer based on GeneMANIA network analysis.

Degree Centrality	Gene Symbol	Official Full Name	Interacting Gene	Networks *
13	ESR1	Estrogen receptor 1	AKT1	2
			AR	2, 3, 5, 6
			BRCA1	2, 3
			CASP8	7
			EP300	2, 3
			ERBB2	2
			HDAC5	2
			NCOA1	2, 3
			NCOA7	2, 3
			PIK3CA	2, 3
			SLC10A1	1
			SMO	1
			TP53	2
12	TP53	Tumor protein p53	AKT1	1
			AR	2, 7
			BCL6	2, 3
			BRCA1	2, 3
			CASP8	2
			EP300	2, 7
			ERBB2	1
			ESR1	2
			HDAC5	2
			MTOR	2
			NCOA1	2
			SMO	1
12	NCOA1	Nuclear receptor coactivator 1	AKT1	1, 3
			AR	2, 3
			BRCA1	2
			CYP1A1	1
			DUSP10	1, 7
			EP300	1, 2, 3, 6
			ESR1	2, 3
			HDAC5	7
			KLHL24	1
			NCOA7	7
			PTCH1	7
			TP53	2
11	AKT1	AKT serine/threonine kinase 1	AR	2
			BRCA1	2
			EP300	2
			ERBB2	1
			ESR1	2
			MAP2K2	1
			MTOR	2, 3, 5, 4
			NCOA1	1, 3
			PIK3CA	2, 3
			SMO	3
			TP53	1

Table S1. *Cont.*

Degree Centrality	Gene Symbol	Official Full Name	Interacting Gene	Networks *
11	BCL6	B-cell CLL/lymphoma 6	ABCG1	7
			APOB	1, 7
			EP300	2, 7
			GABRR1	7
			HDAC5	2, 3
			KLHL24	1
			PIK3CA	1
			PTCH1	7
			SLC10A1	1
			SMO	2
			TP53	2, 3
10	AR	Androgen receptor	AKT1	2
			BRCA1	2, 3
			CASP8	1, 3, 4
			EP300	2
			ESR1	2, 3, 5, 6
			NCOA1	2, 3
			PIK3CA	3
			SLC10A1	1
			SMO	1
			TP53	2, 4
9	EP300	E1A binding protein p300	AKT1	2
			AR	2
			BCL6	2, 7
			BRCA1	2, 7
			ESR1	2, 3
			KLHL24	1
			NCOA1	1, 2, 3, 6
			PIK3CA	1
			TP53	2, 7
9	PIK3CA	Phosphatidylinositol-4,5-bisphosphate 3-kinase catalytic subunit alpha	ABCG1	1
			AKT1	2, 3
			AR	3
			BCL6	1
			EP300	1
			ERBB2	3
			ESR1	2, 3
			KLHL24	1
			MTOR	3, 6
8	SMO	Smoothened, frizzled class receptor	AR	1
			AKT1	3
			BCL6	2
			CYP1A1	1
			ESR1	1
			ERBB2	1
			PTCH1	2, 3
			TP53	1
7	BRCA1	BRCA1, DNA repair associated	AKT1	2
			AR	2, 3
			EP300	2, 7
			ESR1	2, 3
			MTOR	2
			NCOA1	2
			TP53	2, 3
7	CASP8	Caspase 8	AR	2, 3, 4
			CYP1A1	1
			ESR1	7
			GABRA6	7
			KLHL24	7
			MTOR	7
			TP53	2

Table S1. *Cont.*

Degree Centrality	Gene Symbol	Official Full Name	Interacting Gene	Networks *
6	GABRA6	Gamma-aminobutyric acid type A receptor alpha6 subunit	APOB	1
			CASP8	7
			GABRR1	1, 6, 7
			LFNG	1
			PTCH1	7
			SLC10A1	1
6	HDAC5	Histone deacetylase 5	BCL6	2, 3
			ESR1	2
			LFNG	1
			MAP2K2	1
			NCOA1	7
			TP53	2
6	MTOR	Mechanistic target of rapamycin	AKT1	2, 3, 4, 5
			BRCA1	2
			CASP8	7
			MAP2K2	1
			PIK3CA	3, 6
			TP53	2
5	ERBB2	Erb-b2 receptor tyrosine kinase 2	AKT1	1
			ESR1	2
			PIK3CA	3
			SMO	1
			TP53	1
5	GABRR1	Gamma-aminobutyric acid type A receptor rho1 subunit	BCL6	7
			CYP1A1	7
			DUSP10	7
			GABRA6	1, 6, 7
			LFNG	1
5	KLHL24	Kelch like family member 24	BCL6	1
			CASP8	7
			EP300	1
			NCOA1	1
			PIK3CA	1
4	CYP1A1	Cytochrome P450 family 1 subfamily A member 1	CASP8	1
			GABRR1	7
			NCOA1	1
			SMO	1
4	PTCH1	Patched 1	BCL6	7
			GABRA6	7
			NCOA1	7
			SMO	2, 3
4	SLC10A1	Solute carrier family 10 member 1	AR	1
			BCL6	1
			ESR1	1
			GABRA6	1
3	ABCG1	ATP binding cassette subfamily G member 1	APOB	7
			BCL6	7
			PIK3CA	1
3	APOB	Apolipoprotein B	ABCG1	7
			BCL6	1, 7
			GABRA6	1
3	LFNG	LFNG O-fucosylpeptide 3-beta-N-acetylglucosaminyltransferase	GABRA6	1
			GABRR1	1
			HDAC5	1
3	MAP2K2	Mitogen-activated protein kinase kinase 2	AKT1	1
			HDAC5	1
			MTOR	1
2	DUSP10	Dual specificity phosphatase 10	GABRR1	7
			NCOA1	1, 7
2	NCOA7	Nuclear receptor coactivator 7	ESR1	2, 3
			NCOA1	7
0	KIF21B	Kinesin family member 21B	-	-

* 1—Co-expression; 2—Physical interactions; 3—Pathway; 4—Predicted; 5—Co-localization; 6—Shared protein domains; 7—Genetic interactions.

Table S2. The list of candidate EDCs with the score and their interacting genes curated from the CTD.

Score	Chemical Name (Cas No.)	Interacting Genes
40	Perfluorooctanoic acid (335-67-1)	ABCG1, APOB, CYP1A1, ERBB2, ESR1, TP53, SHH
37	Stearic acid (57-11-4)	ABCG1, AKT1, AR, ESR1
35	Triphenyl phosphate (115-86-6)	AR, ESR1, TP53
34	Dibutyl Phthalate (84-74-2)	AKT1, AR, ESR1
30	Sodium Fluoride (7681-49-4)	AKT1, CASP8, TP53, FAS
28	Perfluorodecanoic acid (335-76-2)	AKT1, CYP1A1, ESR1
23	Hydroquinone (123-31-9)	CASP8, CYP1A1, TP53, FAS
23	Butylated Hydroxyanisole (25013-16-5)	AR, ESR1
23	Homosalate (118-56-9)	AR, ESR1
23	Oxybenzone (131-57-7)	AR, ESR1
23	Phantolid (15323-35-0)	AR, ESR1
23	Triclosan (3380-34-5)	AR, ESR1
17	Butylparaben (94-26-8)	CYP1A1, ESR1
14	Lead acetate (301-04-2)	AKT1, MAP2K2
13	2,2',4,4'-tetrahydroxybenzophenone (131-55-5)	ESR1
13	2,4-dihydroxybenzophenone (131-56-6)	ESR1
13	2-tert-butylphenol (88-18-6)	ESR1
13	4-hydroxybenzophenone (1137-42-4)	ESR1
13	Benzophenone (119-61-9)	ESR1
13	Benzylparaben (94-18-8)	ESR1
13	benzyl salicylate (118-58-1)	ESR1
13	Di-n-octyl phthalate (117-84-0)	ESR1
13	Dioxybenzone (131-53-3)	ESR1
13	Ethyl-p-hydroxybenzoate (120-47-8)	ESR1
13	FD & C Yellow No. 6 (2783-94-0)	ESR1
13	Isopropyl 4-hydroxybenzoate (4191-73-5)	ESR1
13	Methylparaben (99-76-3)	ESR1
13	Methyl salicylate (119-36-8)	ESR1
13	Octamethylcyclotetrasiloxane (556-67-2)	ESR1
13	Octocrylene (6197-30-4)	ESR1
13	Perfluoro-n-nonanoic acid (375-95-1)	ESR1
13	Phenyl salicylate (118-55-8)	ESR1
13	Propylparaben (94-13-3)	ESR1
13	Resorcinol (108-46-3)	ESR1
12	Titanium dioxide (13463-67-7)	TP53, FAS
12	Nonidet P-40 (9036-19-5)	TP53
12	Quinoline (91-22-5)	TP53
12	Silver Nitrate (7761-88-8)	TP53
12	Zinc chloride (7646-85-7)	TP53
11	Phloroglucinol (108-73-6)	AKT1
10	2-methylresorcinol (608-25-3)	AR
10	Acetyl methyl tetramethyl tetralin (21145-77-7)	AR
10	Acetyl tert-butyl dimethylindan (13171-00-1)	AR
10	Butylated Hydroxytoluene (128-37-0)	AR
10	Versalide (88-29-9)	AR
7	Catechol (120-80-9)	CASP8, FAS
6	Acetaldehyde (75-07-0)	MTOR
4	1-naphthol (90-15-3)	CYP1A1
4	Isoeugenol (97-54-1)	CYP1A1
4	Piperonyl Butoxide (51-03-6)	CYP1A1
4	Perfluorohexanesulfonic acid (355-46-4)	SLC10A1
3	Aluminum (7429-90-5)	APOB

Table S3. The common 27 genes between EDCs and breast cancer, and evidence showing their relation with breast cancer.

Gene Symbol	Relation with Breast Cancer	References
ABCG1	Significantly upregulated in breast carcinoma tumors compared with control tissues	[1]
AKT1	Mutated in breast cancer	[2]
	Statistically significant exclusion mutation pattern in breast tumors	[3]
APOB	ApoB levels was inversely associated with breast cancer risk	[4]
	rs693 and rs1042031 polymorphisms in the APOB gene increased the risk of breast cancer	[5]
	ApoB levels were negatively associated with breast cancer risk	[6]
AR	AR variant, Δ3AR, has been found exclusively in some breast tumors and breast cancer cell lines but not in normal breast tissue	[7]
BCL6	BCL6 stimulates the oncogenicity of breast cancer cells	[8]
BRCA1	Mutated in breast cancer	[9]
CASP8	CASP8 SNPs showed significant associations with breast cancer	[10]
	Genetic variation is associated with the risk of various cancers	[11]
CYP1A1	CYP1A1 polymorphisms are associated with the risk of breast cancer1	[12]
DUSP10	-	
EP300	Known as breast cancer related genes	[13]
ERBB2	Amplified in 20 to 30% of breast cancers, and overexpression is associated with an aggressive phenotype of tumor	[9]
	Overexpression in 25-30% breast tumors	[14]
ESR1	Over two-thirds of cases in breast cancer express ESR1	[15]
GABRA6	-	
GABRR1	-	
HDAC5	Extensively expressed in breast cancer tissues	[16]
KIF21B	-	
KLHL24	-	
LFNG	Reduced expression in majority of breast basal tumors	[17]
	Overexpression in luminal A breast cancer	[18]
MAP2K2	-	
MTOR	The PI3K/AKT/mTOR pathway alterations play significant roles in breast cancer	[19]
NCOA1	Overexpression positively correlates with breast cancer recurrence and metastasis	[20]
NCOA7	Alterations in gene expression affect breast cancer risk	[21]
	Genetic variants is associated with breast cancer	[22]
PIK3CA	Statistically significant exclusion mutation pattern in breast cancer mutations	[3]
	Mutated in 45% of luminal breast cancer	[23]
PTCH1	Sonic hedgehog/Patched (SHH/PTCH1) signaling pathway is involved in hormone-induced development of breast carcinoma	[24]
SLC10A1	-	
SMO	Overexpression in primary breast cancers	[25]
	Play a role in maintaining breast cancer stem cell features	[26]
TP53	Mutated in 30% of breast cancer	[9]

References in Table S3

1. Hlavac, V.; Brynychova, V.; Vaclavikova, R.; Ehrlichova, M.; Vrana, D.; Pecha, V.; Kozevnikovova, R.; Trnkova, M.; Gatek, J.; Kopperova, D. et al. The expression profile of atp-binding cassette transporter genes in breast carcinoma. *Pharmacogenomics* **2013**, *14*, 515–529.
2. Banerji, S.; Cibulskis, K.; Rangel-Escareno, C.; Brown, K.K.; Carter, S.L.; Frederick, A.M.; Lawrence, M.S.; Sivachenko, A.Y.; Sougnez, C.; Zou, L.H. et al. Sequence analysis of mutations and translocations across breast cancer subtypes. *Nature* **2012**, *486*, 405–409.

3. Koboldt, D.C.; Fulton, R.S.; McLellan, M.D.; Schmidt, H.; Kalicki-Veizer, J.; McMichael, J.F.; Fulton, L.L.; Dooling, D.J.; Ding, L.; Mardis, E.R. et al. Comprehensive molecular portraits of human breast tumours. *Nature* **2012**, *490*, 61–70.
4. Borgquist, S.; Butt, T.; Almgren, P.; Shiffman, D.; Stocks, T.; Orho-Melander, M.; Manjer, J.; Melander, O. Apolipoproteins, lipids and risk of cancer. *Int. J. Cancer* **2016**, *138*, 2648–2656.
5. Liu, X.Y.; Wang, Y.; Qu, H.L.; Hou, M.H.; Cao, W.H.; Ma, Z.L.; Wang, H.B. Associations of polymorphisms of rs693 and rs1042031 in apolipoprotein b gene with risk of breast cancer in chinese. *Jpn. J. Clin. Oncol.* **2013**, *43*, 362–368.
6. Martin, L.J.; Melnichouk, O.; Huszti, E.; Connelly, P.W.; Greenberg, C.V.; Minkin, S.; Boyd, N.F. Serum lipids, lipoproteins, and risk of breast cancer: A nested case-control study using multiple time points. *J. Natl. Cancer Inst.* **2015**, *107*. pii: djv032.
7. Louie, M.C.; Sevigny, M.B. Steroid hormone receptors as prognostic markers in breast cancer. *Am. J. cancer res.* **2017**, *7*, 1617–1636.
8. Yan, H.; Zhao, M.; Huang, S.; Chen, P.; Wu, W.Y.; Huang, J.; Wu, Z.S.; Wu, Q. Prolactin inhibits bcl6 expression in breast cancer cells through a microrna-339-5p-dependent pathway. *J. breast cancer* **2016**, *19*, 26–33.
9. Banin Hirata, B.K.; Oda, J.M.M.; Losi Guembarovski, R.; Ariza, C.B.; Oliveira, C.E.C.d.; Watanabe, M.A.E. Molecular markers for breast cancer: Prediction on tumor behavior. *Dis. markers* **2014**, *2014*, 513158.
10. Cox, A.; Dunning, A.M.; Garcia-Closas, M.; Balasubramanian, S.; Reed, M.W.; Pooley, K.A.; Scollen, S.; Baynes, C.; Ponder, B.A.; Chanock, S. et al. A common coding variant in casp8 is associated with breast cancer risk. *Nat. Genet.* **2007**, *39*, 352–358.
11. Zhang, Y.; Li, W.; Hong, Y.; Wu, G.; He, K.; Liu, D. A systematic analysis of the association studies between casp8 d302h polymorphisms and breast cancer risk. *J. genet.* **2017**, *96*, 283–289.
12. Farzaneh, F.; Noghabaei, G.; Barouti, E.; Pouresmaili, F.; Jamshidi, J.; Fazeli, A.; Emamalizadeh, B.; Darvish, H. Analysis of cyp17, cyp19 and cyp1a1 gene polymorphisms in iranian women with breast cancer. *Asian Pac. J. Cancer Prev.* **2016**, *17*, 23–26.
13. Zhang, Y.; Zhang, J.; Liu, Z.; Liu, Y.; Tuo, S. A network-based approach to identify disease-associated gene modules through integrating DNA methylation and gene expression. *Biochem. Biophys. Res. Commun.* **2015**, *465*, 437–442.
14. Newman, S.P.; Bates, N.P.; Vernimmen, D.; Parker, M.G.; Hurst, H.C. Cofactor competition between the ligand-bound oestrogen receptor and an intron 1 enhancer leads to oestrogen repression of erbb2 expression in breast cancer. *Oncogene* **2000**, *19*, 490–497.
15. Robinson, D.R.; Wu, Y.M.; Vats, P.; Su, F.Y.; Lonigro, R.J.; Cao, X.H.; Kalyana-Sundaram, S.; Wang, R.; Ning, Y.; Hodges, L. et al. Activating esr1 mutations in hormone-resistant metastatic breast cancer. *Nat. Genet.* **2013**, *45*, 1446–1451.
16. Li, A.Q.; Liu, Z.B.; Li, M.; Zhou, S.L.; Xu, Y.; Xiao, Y.X.; Yang, W.T. Hdac5, a potential therapeutic target and prognostic biomarker, promotes proliferation, invasion and migration in human breast cancer. *Oncotarget* **2016**, *7*, 37966–37978.
17. Zhang, S.; Chung, W.C.; Miele, L.; Xu, K. Targeting met and notch in the lfng-deficient, met-amplified triple-negative breast cancer. *Cancer biol. Ther.* **2014**, *15*, 633–642.
18. Orzechowska, M.; Jedroszka, D.; Bednarek, A.K. Common profiles of notch signaling differentiate disease-free survival in luminal type a and triple negative breast cancer. *Oncotarget* **2017**, *8*, 6013–6032.
19. Azim, H.A.; Kassem, L.; Treilleux, I.; Wang, Q.; El Enein, M.A.; Anis, S.E.; Bachelot, T. Analysis of pi3k/mtor pathway biomarkers and their prognostic value in women with hormone receptor-positive, her2-negative early breast cancer. *Transl. oncol.* **2016**, *9*, 114–123.
20. Qin, L.; Xu, Y.; Xu, Y.; Ma, G.; Liao, L.; Wu, Y.; Li, Y.; Wang, X.; Wang, X.; Jiang, J. et al. Ncoa1 promotes angiogenesis in breast tumors by simultaneously enhancing both hif1alpha- and ap-1-mediated vegfa transcription. *Oncotarget* **2015**, *6*, 23890–23904.
21. Sullner, J.; Lattrich, C.; Haring, J.; Gorse, R.; Ortmann, O.; Treeck, O. A polymorphism in the nuclear receptor coactivator 7 gene and breast cancer susceptibility. *Oncol. Lett.* **2012**, *3*, 131–134.
22. Higginbotham, K.S.; Breyer, J.P.; Bradley, K.M.; Schuyler, P.A.; Plummer, W.D., Jr.; Freudenthal, M.E.; Trentham-Dietz, A.; Newcomb, P.A.; Sanders, M.E.; Page, D.L. et al. A multistage association study identifies a breast cancer genetic locus at ncoa7. *Cancer Res.* **2011**, *71*, 3881–3888.

23. Ramirez-Ardila, D.; Timmermans, A.M.; Helmijr, J.A.; Martens, J.W.M.; Berns, E.; Jansen, M. Increased mapk1/3 phosphorylation in luminal breast cancer related with pik3ca hotspot mutations and prognosis. *Transl. Oncol.* **2017**, *10*, 854–866.
24. Chang-Claude, J.; Dunning, A.; Schnitzbauer, U.; Galmbacher, P.; Tee, L.; Wjst, M.; Chalmers, J.; Zemzoum, I.; Harbeck, N.; Pharoah, P.D. et al. The patched polymorphism pro1315leu (c3944t) may modulate the association between use of oral contraceptives and breast cancer risk. *Int. J. Cancer* **2003**, *103*, 779–783.
25. Ge, X.; Lyu, P.; Gu, Y.; Li, L.; Li, J.; Wang, Y.; Zhang, L.; Fu, C.; Cao, Z. Sonic hedgehog stimulates glycolysis and proliferation of breast cancer cells: Modulation of pfkfb3 activation. *Biochem. Biophys. Res. Commun.* **2015**, *464*, 862–868.
26. Wang, L.; Duan, W.; Kang, L.; Mao, J.; Yu, X.; Fan, S.; Li, L.; Tao, Y. Smoothened activates breast cancer stem-like cell and promotes tumorigenesis and metastasis of breast cancer. *Biomed. Pharmacother.* **2014**, *68*, 1099–1104.

© 2017 by the authors. Licensee MDPI, Basel, Switzerland. This article is an open access article distributed under the terms and conditions of the Creative Commons Attribution (CC BY) license (http://creativecommons.org/licenses/by/4.0/).
